# Analysis of frailty and survival from late middle age in the Beijing Longitudinal Study of Aging

**DOI:** 10.1186/1471-2318-11-17

**Published:** 2011-04-20

**Authors:** Jing Shi, Xiaowei Song, Pulin Yu, Zhe Tang, Arnold Mitnitski, Xianghua Fang, Kenneth Rockwood

**Affiliations:** 1 Beijing Institute of Geriatrics, Beijing Hospital, Ministry of Health, Beijing, China; 2 Department of Epidemiology and Social Medicine, Xuanwu Hospital, The Capital Medical University, Beijing, China; 3 Department of Medicine, Dalhousie University, Halifax, Nova Scotia, Canada; 4 National Research Council, Institute for Biodiagnostics-Atlantic, Halifax, Nova Scotia, Canada; 5 Department of Mathematics and Statistics, Dalhousie University, Halifax, Nova Scotia, Canada; 6 Central for Health Care of the Elderly, QEⅡHealth Sciences Centre, Capital District Health Authority, Halifax, Nova Scotia, Canada

## Abstract

**Background:**

Frailty in individuals can be operationalized as the accumulation of health deficits, for which several trends have been observed in Western countries. Less is known about deficit accumulation in China, the country with the world's largest number of older adults.

**Methods:**

This study analyzed data from the Beijing Longitudinal Study of Aging, to evaluate the relationship between age and deficit accumulation in men and women and to evaluate the impact of frailty on mortality. Community dwelling people aged 55+ years at baseline (n = 3275) were followed every two to three years between 1992 and 2000, during which time 36% died. A Frailty Index was constructed using 35 deficits, drawn from a range of health problems, including symptoms, disabilities, disease, and psychological difficulties.

**Results:**

Most deficits increased the eight-year risk of death and were more lethal in men than in women, although women had a higher mean level of frailty (Frailty Index = 0.11 ± 0.10 for men, 0.14 ± 0.12 for women). The Frailty Index increased exponentially with age, with a similar rate in men and women (0.038 vs. 0.039; r > 0.949, P < 0.01). A dose-response relationship was observed as frailty increased.

**Conclusions:**

A Frailty Index employed in a Chinese sample, showed properties comparable with Western data, but deficit accumulation appeared to be more lethal than in the West.

## Background

As populations age, on average, the need for health care increases. Even so, that average increase masks considerable heterogeneity, a topic of increasing relevance to health care planners. Heterogeneity of health and vulnerability to adverse outcomes in people of the same chronological age is commonly referred to as frailty [1].

Despite a growing research literature on frailty, several operational definitions of frailty are employed. Notably, frailty in individuals can be operationalized as a phenotype or as the accumulation of deficits [2-4]. The deficit accumulation approach is based on the observation that as people age, they experience problems which can accumulate. As deficits (symptoms, signs, illnesses, disabilities) accumulate, people become more susceptible to adverse health outcomes, including worse health and even death. Counting deficits allows grades of frailty to be discerned. It also provides insights into the complex problems of older adults, in that, operationalized as deficit accumulation, several replicable trends have been observed in Western countries [5-11]. For example, deficits increase exponentially with age, at about 0.03 per year beginning by at least late middle age [5,12,13]. Women accumulate more deficits than do men, but appear to tolerate them better, because at any level of deficit accumulation, women have a lower mortality rate than men do [14-16]. Mean frailty is associated very highly with mortality [12]. Intriguingly too, there is a limit to frailty - at a frailty index (FI) value of about ~0.7 - after which further deficit accumulation is very unlikely [11,12,17] Deficit accumulation is also associated with a variety of adverse biological features, such as impaired response to vaccination and elevated markers of chronic inflammation [18,19].

In Chinese samples, less is known about how frailty can be assessed. In the Chinese Longitudinal Healthy Longevity Survey (CLHLS), Gu et al. have reported that a FI can be used to robustly predict mortality at advanced ages and that the relationship between frailty and mortality is independent of a number of covariates [10]. In a Hong Kong sample, Goggins et al. demonstrated that the FI increased with age until mid-80s, when it levelled off [6]. A frailty index has also been shown to be significantly related to changes in the total Activities of Daily Living (ADL) score, mental score, and days of hospital stay [20]. Even so, within and between country variations in deficit accumulation remain of interest [21], especially in relation to the slope of the FI with age, its relation to mortality and the presence of a limit. To better understand frailty in China in relation to deficit accumulation, we analyzed data from the Beijing Longitudinal Study of Aging (BLSA) and compared the results with that in the Western populations. The objectives of the study were: 1. To examine the relationship between frailty as the accumulation of deficits and age, comparing this relationship in women and men. 2. To evaluate the relationship between frailty and mortality. 3. To evaluate whether a sub-maximal limit to frailty was present.

## Methods

### Study setting and participants

**Figure 1:**
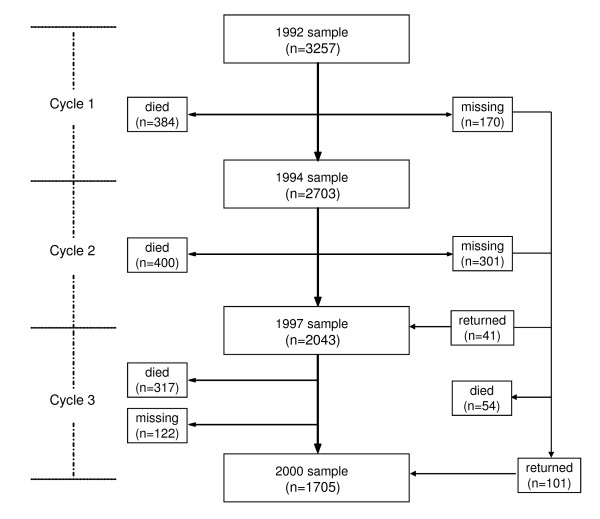
**Composition of Beijing Longitudinal Study of Aging (BLSA) cohort**.

This is a secondary analysis of the Beijing Longitudinal Study of Aging (BLSA), a representative cohort of 3,275 community dwelling Chinese people from late middle age (age 55 years and older). This dataset was provided by the Beijing Geriatric Clinical and Research Centre, Xuanwu Hospital of Capital Medical University, Beijing, China. Distributions of gender, age groups, and educational categories of the study sample represent those of older population in Beijing, as obtained from the Fourth National Census Data [22]. As described elsewhere [22,23], the cohort was assembled in 1992 (The response rate at baseline was 91.2%), and followed every two to three years (cycles 1, 2, and 3) in 1994, 1997 and 2000. Of the 3,257 participants sampled at baseline (1992), 1,593 (48.9%) were men and 1,664 (51.1%) women, with an average age of 70.1 ± 9.0 years. By 2000, 1705 people survived, 1155 had died, and 397 were lost to follow-up (Figure 1). There are no statistically significant differences between respondents to all cycles and those lost to follow-up, with regard to gender, education level and dwelling areas. At each BLSA cycle, a health survey was conducted, using a self-reported questionnaire. Information was obtained at respondents' homes by trained interviewers, usually nurses or doctors. Self-reported information, which covered demographic characteristics, socio-economic status, functional abilities, life style, the use of medical services, physical health including diseases, psychological health, and cognitive status (e.g., the Mini Mental State Examination - MMSE) was collected. The presence of disease was verified in the medical records provided by the subjects. For the present study, variables from the baseline (1992) survey were used to construct the FI, while the eight-year survival outcomes (i.e., in 2000) were evaluated. Survival outcomes were determined through interviews with surviving household members and, when surviving household members were not available, with neighbours.

### Construction of the Frailty Index (FI)

A standard procedure was followed to develop the FI [24]. Individual deficits were included in the FI if they were sensible measures of health (e.g., eye colour would not be included, because while it is a verifiable physical characteristic, it is not a health deficit), accumulated with age, and did not saturate too early (i.e. did not always become a deficit by some relatively young age). In addition, each variable should have >1% prevalence and <5% missing values. The variables which made up the FI covered a range of health problems, including symptoms (n = 7), ADL and Instrumental Activities Daily Living (IADL) disabilities (n = 14), diseases (n = 8), psychological problems (n = 5) and MMSE score (Table three). For the 15 binary variables, the presence of a deficit was coded as "1" and its absence was coded as '0'. For the remaining 20 3-scale variables (20/35), a single intermediate response level (e.g. 'sometimes' or 'maybe') was coded using an additional value of '0.5'. We transformed MMSE score into a 3 level variable coded as 0 if MMSE ≥24, 0.5 for MMSE between 15 and 23 and 1 if MMSE < 15. For missing data the FI was calculated based on the items which were present, i.e. the missing variable was excluded from both the numerator and the denominator. No individual had > 5% missing deficit items.

### Analysis

Demographic data were described using means and standard deviations (for age, total ADL, IADL MMSE, and CES-D scores and the total number of co-morbidities) or percentages (for gender, education level, and dwelling status) and were analyzed using analysis of variance (ANOVA) and chi-square, respectively. In these analyses, the FI results were grouped in increments of 0.1 (including groups combining the FIs less than 0.1 and the FIs larger than 0.5). Univariate logistic regression was used to estimate the likelihood of eight-year mortality for each deficit included in the FI. Nonlinear regression techniques were used to fit age-specific frailty index values as a function of age (an exponential function) and to fit the probability of death as a function of the frailty index (a logistic function) and to estimate the parameters of the models. Survival was evaluated using Kaplan-Meier curves (censoring for non-responders) stratified for different level of the FI. A Cox proportional hazards model was used to evaluate the effect of covariates (age, sex, and frailty) on mortality after testing for the proportionality assumption. The construction and evaluation of nonlinear fits were done using Matlab (version 7.1, Mathworks Inc), otherwise (e.g., survival analysis, logistic regression) SPSS version 15.0 for Windows was used.

The statistical significance level was set as *P * = .05.

### Ethics

Approval for these secondary analyses was granted by the Research Ethics Committee of the Capital District Health Authority, Halifax, Canada, where the data are held and analyses were conducted.

## Results

People who were frailer tended to be older, less educated, more often female and rural dwelling people (Table 1). A similar trend was observed for the oldest older adults (aged 85^+ ^years) compared to younger ones (Table 2).

**Table 1:** Demographic characteristics of the sample as separated by the level of FI

Level of FI	≤**0.1**	0.1-0.2	0.2-0.3	0.3-0.5	>0.5	**F/X**^**2**^
**n**	1,650	1,028	330	211	38	--
**Age**	66.8 ± 7.5	70.9 ± 8.3	75.6 ± 7.8	77.8 ± 7.9	76.8 ± 8.3	182.54**
**Female (%)**	44.9	56.4	61.6	55.7	77.8	63.01**
**9**^**+ **^**year education (%)**	25.7	20.0	12.1	11.3	7.6	57.69**
**Rural dwelling (%)**	19.3	19.6	25.1	30.6	31.5	43.68**
**Total ADL score**	6.0 ± 0.1	6.0 ± 0.2	6.3 ± 0.6	8.5 ± 3.0	14.8 ± 2.5	1535.50**
**Total IADL score**	6.1 ± 0.4	6.9 ± 1.6	9.3 ± 3.2	14.8 ± 2.5	17.8 ± 0.4	2527.3**
**Co-morbidities (count)**	1.4 ± 1.5	1.3 ± 1.4	1.2 ± 1.3	1.1 ± 1.5	1.0 ± 1.4	2.74*
**MMSE score**	24.2 ± 3.7	21.8 ± 4.3	20.4 ± 4.5	17.2 ± 5.3	19.6 ± 2.2	61.38**
**CES-D score**	5.2 ± 5.7	7.4 ± 7.6	11.1 ± 9.1	13.5 ± 9.8	12.4 ± 6.6	55.83**

Note: FI: Frailty Index. MMSE: Mini-Mental Status Examination. CES-D: Center for Epidemiologic Studies Depression Scale. F: Analysis of Variance. X2: Chi-square. Data presented are mean ± standard deviation, otherwise as specified. **: p < 0.01; *: p < 0.05;-: no data.

**Table 2:** Demographic characteristics of the sample as separated by age group

Age group (years)	55-64	65-74	75-84	85-94	> = 95	**F/X**^**2**^
**n**	1,039	1,109	934	170	5	--
**Age**	59.8 ± 2.7	69.5 ± 2.8	79.0 ± 2.7	87.5 ± 2.4	95.8 ± 0.8	7964.79**
**Female (%)**	53.6	47.3	51.7	55.9	80.0	12.26*
**9**^+ ^**year education (%)**	31.0	24.3	10.7	10.0	0.0	138.39**
**Rural dwelling (%)**	20.4	20.7	22.7	18.8	20.0	3.61
**Total ADL score**	6.1 ± 0.6	6.2 ± 1.3	6.5 ± 1.9	7.1 ± 2.4	6.6 ± 0.9	27.44**
**Total IADL score**	6.3 ± 1.5	7.1 ± 2.7	8.8 ± 3.8	11.6 ± 4.5	15.8 ± 2.9	184.325**
**Co-morbidities (count)**	1.4 ± 1.5	1.4 ± 1.5	1.2 ± 1.4	1.1 ± 1.4	1.0 ± 1.0	3.47**
**MMSE score**	24.6 ± 3.4	23.3 ± 4.0	21.5 ± 4.6	19.4 ± 5.1	--	78.57**
**CES-D score**	7.4 ± 7.5	6.8 ± 6.9	6.9 ± 7.6	8.6 ± 8.6	--	1.70

Note: FI: Frailty Index. MMSE: Mini-Mental Status Examination. CES-D: Center for Epidemiologic Studies Depression Scale. F: Analysis of Variance. X2: Chi-square. Data presented are mean ± standard deviation, otherwise as specified. **: p < 0.01; *: p < 0.05;-: no data.

In the univariate logistic regression analyses to evaluate items for inclusion in the FI, most items showed an increased 8-year mortality risk for both men and women, save for self reports of disease diagnoses (Table 3). Several of the latter, including stroke, hypertension, thyroid disease, became less common after age 70. Individual deficits were more often associated with death in men than in women. Even so, for the majority of the items, more women reported deficits.

**Table 3:** Occurrence of the individual deficits and their odds radio for 8-year death

	Men		Women	
	
Variable Description	Prevalence (%)	Odd Ratio (95% C.I.)	Prevalence (%)	Odd Ratio (95% C.I.)
**Psychological problems**				
don't have much energy	53.9	2.24 (1.79-2.80)**	64.5	1.80 (1.41-2.28) **
fell less useful	54.8	2.62 (2.09-3.28) **	70.3	2.10 (1.62-2.72) **
don't feel a lot of fun in life	37.4	1.37 (1.10-1.71) **	38.6	1.17 (0.94-1.46)
don't feel very happy	19.6	1.87 (1.43-2.43) **	23.5	1.95 (1.51-2.50) **
feel nothing to do	18.9	2.02 (1.55-2.63) **	24.9	1.71 (1.35-2.18) **
**Diseases**				
hypertension	18.9	1.17 (0.90-1.51)	21.5	0.83 (0.65-1.07)
coronary heart disease	14.7	0.96 (0.72-1.28)	16.3	0.79 (0.60-1.05)
stroke	7.0	2.02 (1.37-2.97) **	4.3	2.86 (1.77-4.63) **
TIA/small stroke	1.9	0.67 (0.31-1.47)	1.3	1.39 (0.59-3.26)
arthritis	5.5	0.87 (0.55-1.37)	7.7	0.56 (0.37-0.86) **
thyroid disease	0.6	1.67 (0.48-5.78)	1.7	0.66 (0.28-1.56)
glaucoma	1.8	1.01 (0.48-2.16)	2.9	0.90 (0.49-1.68)
cataract	11.4	0.86 (0.63-1.20)	12.3	1.12 (0.82-1.52)
**Symptoms**				
urinary incontinence	10.8	2.71 (1.96-3.74) **	27.8	1.65 (1.32-2.06) **
falls	8.3	2.31 (1.61-3.31) **	13.6	1.80 (1.36-2.40) **
fracture	5.6	1.80 (1.17-2.75) **	8.9	1.12 (0.79-1.60)
tremor	7.3	1.64 (1.12-2.39) *	7.1	1.30 (0.88-1.91)
don't hear clearly	21.9	2.98 (2.33-3.80) **	17.6	3.41 (2.63-4.41) **
wear a hearing aid	2.0	0.55 (0.24-1.23)	1.0	1.09 (0.40-2.96)
use a walking stick	17.2	7.34 (5.44-9.91) **	21.3	7.20 (5.55-9.33) **
**ADL and IADL disabilities**				
need help with eating	1.6	12.60 (3.75-42.26) **	2.1	16.30 (5.72-46.41)**
need help with grooming	1.9	8.98 (3.43-23.50) **	2.6	21.40 (7.62-60.12)**
need help with dressing	2.3	11.16 (4.33-28.81) **	2.6	16.64 (6.52-42.47)**
need help with getting on/off bed	2.3	10.80 (4.17-27.92) **	3.1	20.37 (8.05-51.52)**
need help with bathing	6.5	9.77 (5.74-16.60) **	9.8	8.17 (5.59-11.94) **
need help with moving in house	2.7	13.40 (5.24-34.24) **	3.8	13.14 (6.44-26.81)**
need help with cooking meals	18.6	7.48 (5.60-10.00) **	17.0	7.88 (5.91-10.52) **
need help with managing money	10.5	7.45 (5.07-10.95) **	16.4	6.28 (4.73-8.33) **
need help with taking a bus	18.3	7.72 (5.75-10.36) **	36.4	5.33 (4.28-6.65) **
need help with shopping	12.2	8.25 (5.73-11.90) **	20.3	5.96 (4.63-7.75) **
need help with walking 300 meters	8.3	7.33 (4.77-11.28) **	16.0	6.37 (4.79-8.49) **
need help with up/down stairs	10.9	8.97 (6.04-13.31) **	19.5	7.91 (6.03-10.38)**
need help in running housework	51.0	2.31 (1.87-2.84) **	39.2	3.52 (2.85-4.36) **
need any other personal care	4.3	9.58 (4.99-18.41) **	6.4	6.63 (4.23-10.40)**
**MMSE scores**	33.7	3.63 (2.73-4.83) **	62.6	4.06 (2.89-5.71) **

Note: MMSE: Mini-Mental Status Examination. **P < 0.01; *P < 0.05. C.I.: confidence interval.

**Figure 2:**
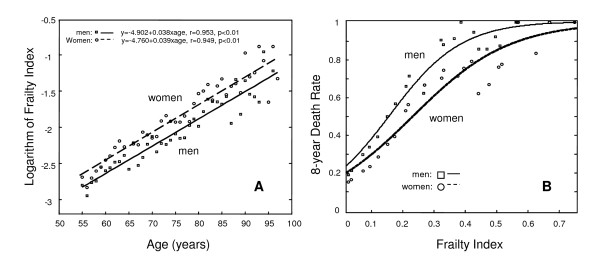
**The relationship between age and the mean value of the FI (Panel A) and the eight-year death rate as a function of the FI (Panel B)**. Men: squares and solid line; women: circles and dashed line.

Most people (over 73%) had FI values between 0-0.15, with a mean value of 0.11 ± 0.10 (median = 0.09) for men and a mean value of 0.14 ± 0.12 (median = 0.10) for women. For both men and women, the mean value of the FI increased exponentially with age, with similar rates for men (0.038) and women (0.039) (Figure 2, Panel A). The correlation coefficient between age and the logarithm of the FI was high for both men (*r *= 0.953, *P *< 0.01) and women (*r *= 0.949, *P *< 0.01).

**Figure 3:**
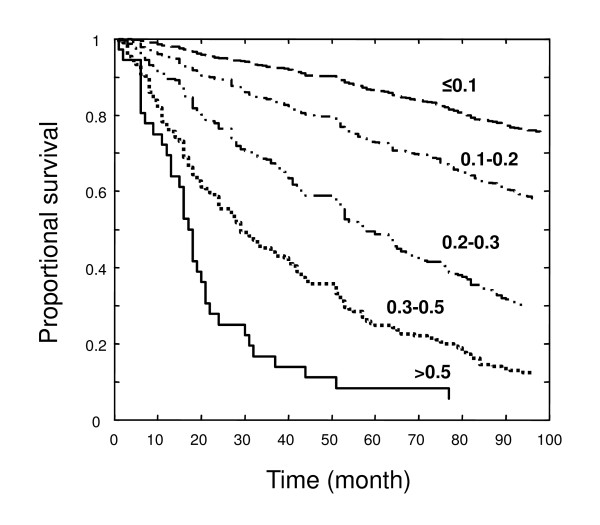
**Kaplan Meier curves for the proportional survival of people with various levels of the FI**. From top to bottom, FI ≤ 0.1 (n = 1510; dashed line), 0.1 < FI ≤ 0.2 (n = 937; dot-dashed line), 0.2 < FI ≤ 0.3 (n = 306; dot-dot-dashed line), 0.3 < FI ≤ 0.5 (n = 193; dotted line), FI > 0.5 (n = 36; solid line).

The FI was highly related to mortality, which was greater in men than in women (Figure 2, Panel B). In other words, although women had more deficits than did men, the deficits were less lethal. For both men and women, increasing grades of the FI showed a dose-response effect in relation to survival, with >90% mortality of the most frail (FI > 0.5) by three and a half years (Figure 3) whereas the total mortality over the entire observation for the least frail (FI < 0.1) was only 18%.

In the proportional hazards analyses, adjusted for sex and age, the number of deficits was more closely related to survival than was age. Every additional deficit used to calculate the FI was associated with an increased hazard rate of 1.13 (95% confidence interval (C.I.) 1.09 to 1.47) on average, compared with the age increment (hazard rate = 1.09; 95% C.I. = 1.06 to 1.10). For example, compared with a 55 year old man with no deficits at baseline, a 75 year old man whose FI = 0.29 (i.e. 10 deficits present of the 35 considered) would have an increased risk of death within 8 years of 5.08. The increased relative risk arising from age would be 1.80 (i.e. 20 year-increments × 1.09); the increased relative risk in relation to frailty would be 3.28 (29 deficit-increments times 1.13).

## Discussion

This study analyzed the health status of older Chinese men and women in the Beijing Longitudinal Study of Aging. From late middle age (55+ years) on, frailty, understood as variable vulnerability of adverse outcomes of people of the same age, could be defined in relation to deficit accumulation. As has been shown by multiple studies from Western countries [8,12], this study further demonstrates that, in a representative Chinese sample, by simply counting the number of deficits present (without considering much of the nature of the deficits), the FI can describe health status and the risk of adverse outcomes. The older an individual person is, the more deficits, on average they will accumulate. Even so, for people of the same age, deficit accumulation is more closely linked to mortality than age is. In other words, while it remains true that the older someone is, the more likely they are to die, this is because the older someone is, the more likely they are to have accumulated deficits, and it is this deficit accumulation, as measured by the FI, which most defines their risk of death.

Of some interest is the convex shape of the mortality curve in relation to the FI, compared with the concave shape which has been observed in the Western countries. This suggests that all levels of deficit accumulation are more lethal in China than in the west. It also suggests that deficit accumulation is only part of the story with regard to variable vulnerability to adverse outcomes - it is also essential to consider the environment in which deficit accumulation occurs - i.e. the deficit model shows the level of insults, but the impact of these insults relies on other factors.

Our data must be interpreted with caution. The BLSA relied on self-report information and it is arguable such data may not be as accurate as clinical examinations. On the other hand, the frailty estimates are similar to what has been discovered for other countries. For example, the increase in the mean value of the FI (0.038; 95% CI = 0.035-0.041) is close to (if somewhat higher than) the mean slope of 0.029 (95% CI = 0.027-0.030) reported for Western countries [5]. In addition, different datasets of the Chinese population revealed similar results. For example, in the Chinese Longitudinal Healthy Longevity Survey, frailty increased with age in both women and men, and women were frailer than men at all ages. Also, the mean value of FI for women was approximately 0.10 at age 65 and increased to 0.15 at age 80 [10], comparable with the values found in the BLSA dataset (0.11 at age 65 and 0.18 at age 80). Given the importance of slow walking speed as a readily clinically observable feature which helps to stratify risk [25,26], and which is reasonably easily measured, it would have been preferable to have this information. Similarly, we do not have a measure of grip strength either, which is also associated with variable mortality and is a key component of the phenotypic definition of frailty [27,28]. On the other hand, many past studies have not used performance measures, so that obliging their presence to speak of frailty means that much information will be lost. In addition, while such items might be preferable, it is not clear that the performance information is essential. Indeed, three groups which have compared the phenotype and deficit accumulation operational definitions have found in each case that the latter stratifies risk at least as well as the former [7,19,29]. Note too that the FI includes aspects of cognition and the mental state which are also important in defining the risk of adverse health outcomes [2-4].

Note too that here we did not weight the deficits which made up the FI. Some groups see this as essential and point out that they can improve the (retrospective) predictive power of any FI in any given dataset by differential weighting [30]. We have recognized this for some time [31], but have chosen not to weight items because of the greater generalizability which comes from using unweighted ones, including the remarkable observation, replicated here, of an empirical limit to frailty of about 0.7. From a measurement standpoint, this suggests that the FI does not have a ceiling effect. With regard to how deficit accumulation operates, the presence of an upper limit is of considerable interest, as it allows the evaluation of physiological redundancy in relation to frailty [32].

People who were frailer tended to be older, less educated, more often female and rural dwelling people (Table 1). Not surprisingly, given that these impairments were included in the FI, they also had higher ADL and IADL scores (Table 1). Of note however, people with a higher FI score tended to have higher CES-D scores (indicating more depressive features) and lower MMSE scores (indicating worse cognition).

It was curious to us that frailer elderly people on average reported fewer co-morbidities than the fit ones, as this is outside of our experience with other FI calculations and therefore unexpected (Table 1). The reason for this is not clear. One reason might be a survivor effect, in that people with more co-morbidities simply did not survive to be included in the sample. However, this appears to be less likely, considering that a large portion of subjects were within 55-80. More likely, the observation represents some combination of reliance on self-report, and surveying a sample which historically had less access to diagnostic services. Many older participants might not have had the opportunity to visit a physician and thus lacked medical records on diseases. Previous research has revealed similar results [28] and a complex relationship between frailty and chronic diseases has been suggested [2].

These analyses were undertaken as part of the Canada-China Collaboration on Aging and Longevity, which has been designed to address frailty in relation to population aging, and to the use of health care services [33,34]. This initial report suggests that the construct of frailty as deficit accumulation is valid in China, in keeping with work conducted in Hong Kong [6,20] and data from the Chinese Longitudinal Healthy Longevity Survey [9,10]. In contrast to the report from Hong Kong, a levelling off of the FI at higher ages has not been found in BLSA or CLHLS [10], something which remains unique in the Hong Kong sample [6]. Although other Chinese datasets have provided a great deal of information about frailty, so far the parameter estimates of age-association of the FI have not been reported. The much greater mortality associated with deficit accumulation is of interest, and is motivating additional inquiries by our group.

## Conclusions

A Frailty Index employed in a Chinese sample showed comparable properties as with Western data, but deficit accumulation in the Chinese sample appeared to be more lethal than in the West.

## Abbreviations

ADL: Activities Daily Living; CES-D: Centre for Epidemiologic Studies Depression Scale, FI: Frailty Index; IADL: Instrumental Activities Daily Living; MMSE: Mini-Mental State Examination.

## Competing interests

The authors declare that they have no competing interests.

## Authors' contributions

JS prepared the data, performed statistical analysis and co-drafted the initial manuscript. XS helped with the analysis design and analysis, and assisted with result interpretation and manuscript preparation. PY appraised review of the initial manuscript and assisted in preparing the data. ZT and XF appraised review of the initial manuscript and collected data. AM designed and assisted data analysis and result interpretation, and revised the manuscript. KR initiated and designed the study, drafted parts of the discussion, revised the paper, assisted in interpreting the results and finally approved the version to be published. All authors read and approved the final version of the manuscript.

## Pre-publication history

The pre-publication history for this paper can be accessed here:

http://www.biomedcentral.com/1471-2318/11/17/prepub

## References

[B1] Rockwood K Fox RA Stolee P Robertson D Beattie BL Frailty in elderly people: an evolving concept CMAJ 1994 150 489 495 8313261 PMC1486322

[B2] Bergman H Ferrucci L Guralnik J Hogan DB Hummel S Karunananthan S Wolfson C Frailty: an emerging research and clinical paradigm--issues and controversies J Gerontol A Biol Sci Med Sci 2007 62 731 737 10.1093/gerona/62.7.731 17634320 PMC2645660

[B3] de Vries NM Staal JB van Ravensberg CD Hobbelen JS Olde Rikkert MG Nijhuis-van der Sanden MW Outcome instruments to measure frailty: A systematic review Ageing Res Rev 2011 10 104 114 10.1016/j.arr.2010.09.001 20850567

[B4] Abellan van Kan G Rolland Y Houles M Gillette-Guyonnet S Soto M Vellas B The assessment of frailty in older adults Clin Geriatr Med 2010 26 275 286 10.1016/j.cger.2010.02.002 20497846

[B5] Mitnitski A Song X Skoog I Broe GA Cox JL Grunfeld E Rockwood K Relative fitness and frailty of elderly men and women in developedcountries andtheir relationship with mortality J Am Geriatr Soc 2005 53 2184 2189 10.1111/j.1532-5415.2005.00506.x 16398907

[B6] Goggins WB Woo J Sham A Ho SC Frailty index as a measure of biological age in a Chinese population J Gerontol A Biol Sci Med Sci 2005 60 1046 1051 10.1093/gerona/60.8.1046 16127111

[B7] Kulminski AM Ukraintseva SV Akushevich IV Arbeev KG Yashin AI Cumulative index of health deficiencies as a characteristic of long life J Am Geriatr Soc 2007 55 935 940 10.1111/j.1532-5415.2007.01155.x 17537097 PMC1893090

[B8] Kulminski AM Ukraintseva SV Kulminskaya IV Arbeev KG Land K Yashin AI Cumulative deficits better characterize susceptibility to death in elderly people than phenotypic frailty: lessons from the Cardiovascular Health Study J Am Geriatr Soc 2008 56 898 903 10.1111/j.1532-5415.2008.01656.x 18363679 PMC2703425

[B9] Dupre ME Gu D Warner DF Yi Z Frailty and type of death among older adults in China: prospective cohort study BMJ 2009 338 b1175 10.1136/bmj.b1175 19359289 PMC2667569

[B10] Gu D Dupre ME Sautter J Zhu H Liu Y Yi Z Frailty and mortality among Chinese at advanced ages J Gerontol B Psychol Sci Soc Sci 2009 64 279 289 19196691 10.1093/geronb/gbn009 PMC2655172

[B11] Armstrong JJ Stolee P Hirdes JP Poss JW Examining three frailty conceptualizations in their ability to predict negative outcomes for home-care clients Age Ageing 2010 39 755 758 10.1093/ageing/afq121 20858672

[B12] Rockwood K Mitnitski A Frailty defined by deficit accumulation and geriatric medicine defined by frailty Clin Geriatr Med 2011 27 17 26 10.1016/j.cger.2010.08.008 21093719

[B13] Mitnitski A Song X Rockwood K Improvement and decline in health status from late middle age: modeling age-related changes in deficit accumulation ExpGerontol 2007 42 1109 1115 10.1016/j.exger.2007.08.002 17855035

[B14] Mitnitski AB Song X Rockwood K The estimation of relative fitness and frailty in community-dwelling older adults using self-report data J Gerontol A Biol Sci Med Sci 2004 59 M627 M632 10.1093/gerona/59.6.M627 15215283

[B15] Graham JE Snih SA Berges IM Ray LA Markides KS Ottenbacher KJ Frailty and 10-year mortality in community-living Mexican American older adults Gerontology 2005 55 644 651 10.1159/000235653 PMC2783319 19690395

[B16] Song X Mitnitski A Rockwood K Prevalence and 10-year outcomes of frailty in older adults in relation to deficit accumulation J Am Geriatr Soc 2010 58 681 687 10.1111/j.1532-5415.2010.02764.x 20345864

[B17] Rockwood K Mitnitski A Limits to deficit accumulation in elderly people Mech Ageing Dev 2006 127 494 496 10.1016/j.mad.2006.01.002 16487992

[B18] Ridda I Macintyre CR Lindley RI A qualitative study to assess the perceived benefits and barriers to the pneumococcal vaccine in hospitalised older people Vaccine 2009 27 3775 3779 10.1016/j.vaccine.2009.03.075 19464561

[B19] Hubbard RE Woodhouse KW Frailty, inflammation and the elderly Biogerontology 2010 11 635 641 10.1007/s10522-010-9292-5 20571864

[B20] Woo J Goggins W Sham A Ho SC Public health significance of the frailty index Disabil Rehabil 2006 28 515 521 10.1080/09638280500215867 16513584

[B21] Oksuzyan A Crimmins E Saito Y O'Rand A Vaupel JW Christensen K Cross- national comparison of sex differences in health and mortality in Denmark, Japan and the US Eur J Epidemiol 2010 25 471 480 10.1007/s10654-010-9460-6 20495953 PMC2903692

[B22] Jiang J Tang Z Meng XJ Futatsuka M Demographic determinants for change in activities of daily living: a cohort study of the elderly people in Beijing J Epidemiol 2002 12 280 286 10.2188/jea.12.280 12164333 PMC10499483

[B23] Tang Z Wang HX Meng C Wu XG Kzerstin E Bengt W Pei JJ The prevalence of functional disability in activities of daily living and instrumental activities of daily living among elderly Beijing Chinese Archives of Gerontology and Geriatrics 1999 29 115 125 10.1016/S0167-4943(99)00026-6 15374065

[B24] Searle SD Mitnitski A Gahbauer EA Gill TM Rockwood K A standard procedure for creating a frailty index BMC Geriatr 2008 8 24 10.1186/1471-2318-8-24 18826625 PMC2573877

[B25] Rothman MD Leo-Summers L Gill TM Prognostic significance of potential frailty criteria J Am Geriatr Soc 2008 56 2211 2116 10.1111/j.1532-5415.2008.02008.x 19093920 PMC2782664

[B26] Dumurgier J Elbaz A Ducimetière P Tavernier B Alpérovitch A Tzourio C Slow walking speed and cardiovascular death in well functioning older adults: prospective cohort study BMJ 2009 339 b4460 10.1136/bmj.b4460 19903980 PMC2776130

[B27] Ling CH Taekema D de Craen AJ Gussekloo J Westendorp RG Maier AB Handgrip strength and mortality in the oldest old population: the Leiden 85-plus study CMAJ 2010 182 429 435 10.1503/cmaj.091278 20142372 PMC2842834

[B28] Fried LP Tangen CM Walston J Newman AB Hirsch C Gottdiener J Seeman T Tracy R Kop WJ Burke G McBurnie MA Cardiovascular Health Study Collaborative Research Group Frailty in older adults: evidence for a phenotype J Gerontol A Biol Sci Med Sci 2001 56 M146 M156 10.1093/gerona/56.3.M146 11253156

[B29] Rockwood K Andrew M Mitnitski A A comparison of two approaches to measuring frailty in elderly people J Gerontol A Biol Sci Med Sci 2007 62 738 743 10.1093/gerona/62.7.738 17634321

[B30] Kamaruzzaman S Ploubidis GB Fletcher A Ebrahim S A reliable measure of frailty for a community dwelling older population Health Qual Life Outcomes 2010 8 123 10.1186/1477-7525-8-123 21029450 PMC2988728

[B31] Song X Mitnitski A MacKnight C Rockwood K Assessment of individual risk of death using self-report data: an artificial neural network compared with a frailty index J Am Geriatr Soc 2004 52 1180 1184 10.1111/j.1532-5415.2004.52319.x 15209659

[B32] Rockwood K Rockwood MR Mitnitski A Physiological redundancy in older adults in relation to the change with age in the slope of a frailty index J Am Geriatr Soc 2010 58 318 323 10.1111/j.1532-5415.2009.02667.x 20370858

[B33] Rockwood K Song X Mitnitski A Yu PL Geriatric medicine and the care of frail elderly people Chin J Geriatr 2009 28 353 365

[B34] Song X Mitnitski A Shi J Yu PL Fang XH Tang Z Rockwood K Quantification of health and ageing in Canada and China: a Canada-China joint health research initiative project Chin J Geriatr 2009 28 793 802

